# Low-density lipoprotein: a versatile nanoscale platform for targeted delivery

**DOI:** 10.1039/d2na00883a

**Published:** 2023-01-24

**Authors:** Luyao Dai, Shuaijun Li, Qian Hao, Ruina Zhou, Hui Zhou, Wenxi Lei, Huafeng Kang, Hao Wu, Yuanpei Li, Xiaobin Ma

**Affiliations:** a Department of Oncology, The Second Affiliated Hospital, Medical School of Xi'an Jiaotong University Xi'an Shaanxi 710061 China; b Department of Biochemistry and Molecular Medicine, UC Davis Comprehensive Cancer Center, University of California Davis Sacramento CA 95817 USA; c Department of Biophysics, School of Basic Medical Sciences, Key Laboratory of Environment and Genes Related to Diseases, Xi'an Jiaotong University Health Science Center Xi'an Shaanxi 710061 China

## Abstract

Low-density lipoprotein (LDL) is a small lipoprotein that plays a vital role in controlling lipid metabolism. LDL has a delicate nanostructure with unique physicochemical properties: superior payload capacity, long residence time in circulation, excellent biocompatibility, smaller size, and natural targeting. In recent decades, the superiority and feasibility of LDL particles as targeted delivery carriers have attracted much attention. In this review, we introduce the structure, composition, advantages, defects, and reconstruction of LDL delivery systems, summarize their research status and progress in targeted diagnosis and therapy, and finally look forward to the clinical application of LDL as an effective delivery vehicle.

## Abstract 2

This review introduces low-density lipoprotein (LDL) delivery systems, summarizes the research status and progress in targeted diagnosis and therapy, and looks forward to the clinical application of LDL as an effective delivery vehicle.

## Graphical abstract

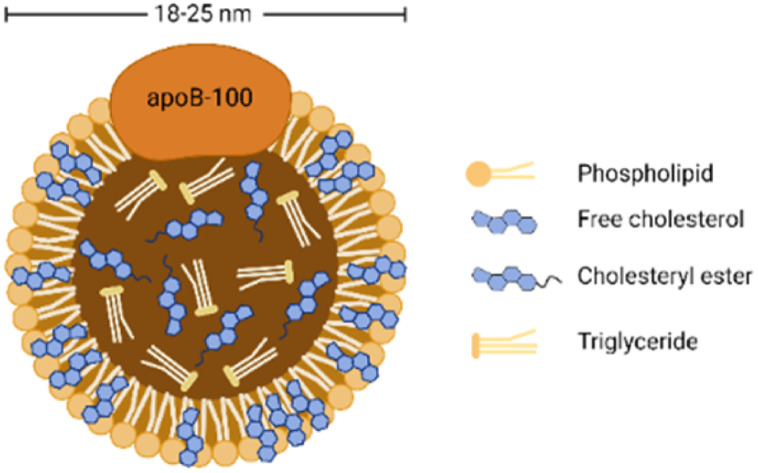

## 1. Introduction

The development and application of nanotechnology have made remarkable progress in the medical field. Various nanoscale building blocks provided alternative delivery options for diagnosing and treating diseases.^1–4^ The Food and Drug Administration (FDA) has approved several nanocarriers for clinical imaging and treatment of cancer or other diseases, such as liposome and lipid-based nanoparticles, protein nanoparticles, polymeric micelles, inorganic nanoparticles, and so on.^5–8^ However, most nanocarriers were trapped in preclinical research for many reasons: difficulties in batch synthesis, biocompatibility problems, lack of suitable targeted selective sites, and especially potential immunotoxicity.^9,10^ An ideal nanocarrier should have excellent biocompatibility, loading efficiency, and targeting ability. Since natural nanoparticles based on lipoproteins can meet these requirements, it is a promising direction of nanomedicine.^11^

So far, the research on the structure and function of lipoproteins has made remarkable progress.^12^ The interior of lipoprotein has abundant hydrophobic triglycerides and cholesterols, indicating that the lipoprotein has large potential to deliver various hydrophobic bioactive compounds such as amphotericin B, cyclosporine A, and halofantrine.^13^ The lipoprotein's outer layer comprises monolayer amphiphilic phospholipids and several apolipoproteins, and the lipoprotein is shown as a spherical nanocomplex that can be transported in the blood. This extended blood circulation property of the lipoprotein allows good systemic administration without additional modification of polyethylene glycol.^14,15^ Besides, lipoproteins are endogenous nanoparticles that would not be regarded as foreign substances by the body's immune system nor be absorbed by the reticuloendothelial system.^16^ Therefore, they have good biocompatibility and limited biological toxicity. In addition, lipoproteins target actively mainly by recognizing the specific receptor of apolipoproteins. These receptors are highly overexpressed on a variety of cancer cells. Thus, the potential ability of lipoprotein targeted delivery is generated by reducing adverse cargo interactions in normal cells and increasing cargo concentration in cancer cells.^17^

The density of plasma lipoproteins is different due to protein and lipid composition ratios. Lipoproteins can be divided into five categories by ultracentrifugation: chylomicrons, very low-density lipoprotein (VLDL), low-density lipoprotein (LDL), intermediate-density lipoprotein (IDL) and high-density lipoprotein (HDL).^18^ The general characteristics of various lipoproteins are shown in Table 1. Among them, LDL and HDL can diffuse into the interfibrillar opening of solid tumors due to their smaller size, so they are widely studied and applied as delivery carriers.^19^ Since the mid-1990s, HDL-based nanovesicles have been widely explored,^20–22^ and the coverage of the reports has significantly exceeded that of LDL.^23^ This review summarizes the latest content on targeted delivery using LDL. Specifically, we dissected the structure and function of LDL and the interaction of LDL receptors. Then, we illustrated the advantages and defects of using LDL as a natural nanocarrier and discussed the common strategies for LDL reconstruction. After that, we cited many examples highlighting LDL nanocarriers' diagnostic and therapeutic capabilities for different diseases. Finally, the summary and prospect of the versatile LDL are put forward.

**Table 1:** Basic characteristics of five types of plasma lipoproteins^24,25^^a^

	Diameter (nm)	Density (g cm^−3^)	Molecular weight (×10^6^ Da)	Apolipoprotein	Composition (wt%)			
					Protein	Cholesterol	Phospholipid	Triglyceride
Chylomicron	75–1200	<0.95	400	ApoA-I, ApoA-II, ApoA-IV, ApoB-48, ApoC-II, ApoE	1–2	2–4	7–9	80–85
VLDL	30–80	0.95–1.006	10–80	ApoB-100, ApoC, ApoE	8–10	17–27	17–19	45–53
LDL	18–25	1.006–1.019	2.3	ApoB-100	20–25	43–50	19–21	5–9
IDL	25–35	1.019–1.063	5–10	ApoB-100, ApoC, ApoE	19	23	20	23
HDL	5–12	1.063–1.210	0.17–0.36	ApoA-I, ApoA-II, ApoC, ApoE	50–60	12–25	17–24	2–3

a Apo, apolipoprotein; VLDL, very low-density lipoprotein; LDL, low-density lipoprotein; IDL, intermediate-density lipoprotein; HDL, high-density lipoprotein.

## 2. LDL and its receptor

### 2.1 Structure and function of LDL

**Fig. 1:**
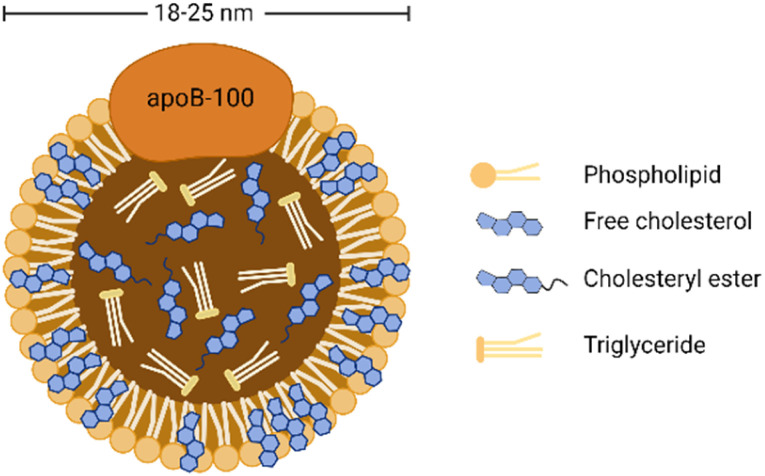
General structure diagram of LDL.

As shown in Fig. 1, LDL is spherical biological nanoparticles with an average diameter of 18–25 nm. Its interior is a hydrophobic core (cholesterol ester and triglyceride). And its exterior is an amphiphilic shell (phospholipid and free cholesterol) with B-100 apolipoprotein molecules (ApoB-100).^26^ Among the components contained in LDL, free and esterified cholesterol account for the most, and triglycerides account for the least. Free cholesterol is inserted between the fatty acid chains of phospholipids, which increases the rigidity of the outer layer of LDL to a certain extent.^27^ ApoB-100 consists of 4536 amino acid residues and covers the particle surface through complex amphiphilic α-helix protein lipid interactions to stabilize this nanostructure.^28^ In addition, another critical role of ApoB-100 is to specifically recognize tissue sites that express LDL receptors (LDLR), such as adrenal glands, skeletal muscles, lymphocytes, gonads, and kidneys.^29^

LDL is the primary cholesterol carrier in blood circulation, and its most important role is to transport cholesterol to extrahepatic tissues for steroid production and membrane synthesis. Cholesterol is an essential component of cell membranes and can be obtained from dietary sources, *de novo* synthesis, or ingestion of circulating low-density lipoprotein.^30^ Although cholesterol is vital for membrane and hormone synthesis, excessive cholesterol supply in peripheral tissues triggers pathological processes such as hyperlipidemia and atherosclerotic plaque formation.^29^ In addition, due to the increased demand of cancer cells for lipids to synthesize new membranes for rapid proliferation, abnormal regulation in cholesterol levels is also associated with multiple types of tumorigenesis.^31^ Many studies have shown that the plasma cholesterol level of cancer patients is reduced and may return to normal after successful cancer treatment.^32–34^ Other studies have pointed out that hypocholesterolemia may be caused by increased LDLR expression in malignant tumors,^35,36^ especially acute myeloid leukemia, rectal cancer, adrenal cancer, lung cancer, liver cancer, brain cancer, metastatic prostate cancer cells, *etc.*^37^

### 2.2 Binding to LDLR

LDLR is a membrane mosaic glycoprotein containing 839 amino acids and can be divided into five independent domains. As mentioned earlier, it is distributed in many different tissues and organs of the body.^38^ By contrast, LDLR is relatively abundant in the liver and kidney to promote their normal uptake and utilization of LDL.^39^ Therefore, although the upregulation of LDLR expression in tumor tissues is conducive to the targeted delivery of LDL, the LDLR expression in normal organs affects this specific tumor-targeting effect. Therefore, reducing the side effects in normal organs is the key to improving targeting efficiency. On the one hand, researchers have identified several specific regulators to inhibit the LDLR activity in normal organs, including bile acid,^40^ sodium taurolaurate and hydrocortisone sodium succinate,^41^ saturated fats,^40^ cholesterol with hydrogenated coconut oil,^42^ modified LDL^43^ (acetylated LDL, methylated LDL and oxidized LDL), and angiotensin-II inhibitors.^44^ On the other hand, alternative receptors for ApoB-100 ligands can also reduce the binding of LDL to normal tissues.^16,45^ The study showed that the alkylation of the side chain of ApoB-100 lysine abolished the LDLR binding activity. Therefore, LDL can be redirected to targeted receptors other than LDLR.^46^

**Fig. 2:**
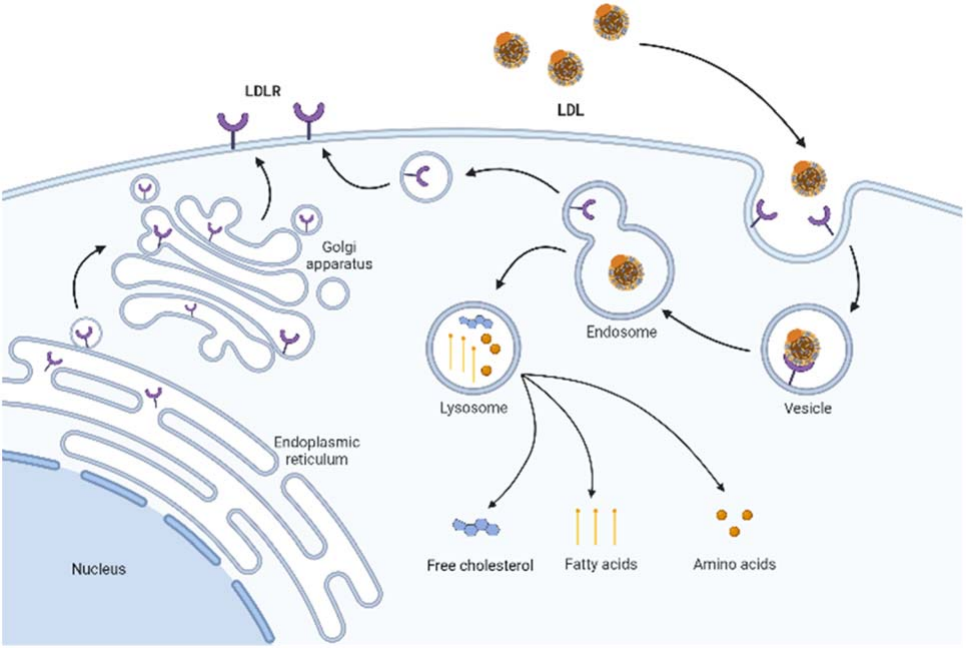
Schematic diagram of LDLR mediated endocytosis.

The cell uptake of LDL with cargo is called LDLR-mediated endocytosis.^47^ This specific process is as follows (Fig. 2). Firstly, LDLR on the extracellular membrane specifically recognizes and embeds ApoB-100 of the LDL's phospholipid monolayer. Then, LDL binds to the ligand binding domain of LDLR and forms a clathrin-coated pit in the cell membrane. Subsequently, the vesicle envelopes with LDL and LDLR invaginate and form a vesicle. After continuous maturation, the vesicle undergoes depolymerization and fusion and is transformed into acidic endosomes. The low pH in the endosomes (as low as pH 5) triggers the separation of LDL from receptors. LDL is further transported into the lysosome and degraded into free cholesterol, fatty acids, and amino acids for use by cells. Finally, the receptor is recycled back to the cell surface and continues to bind and internalize with other LDL.^48^ The turnover time of LDLR is about 24 hours.^49^

## 3. LDL as a versatile nanocarrier

### 3.1 Methods for cargo carrying

**Fig. 3:**
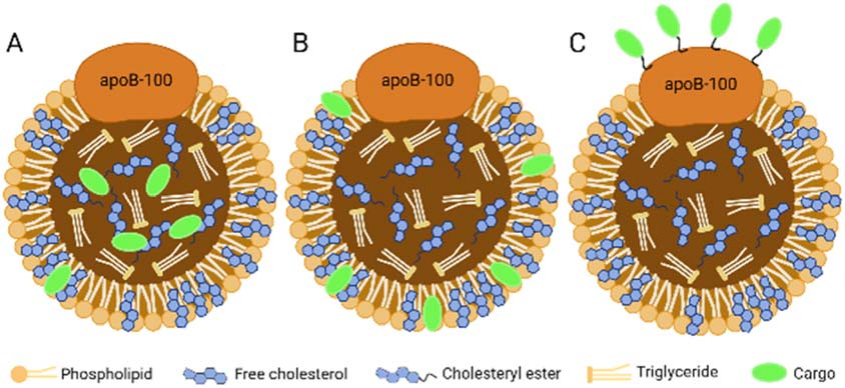
Three methods for cargo carrying in LDL. (A) Loading in the hydrophobic core; (B) inserting into the phospholipid monolayer; (C) binding to the apolipoprotein.

The hydrophobic core of LDL can accommodate lipophilic payload. The amphiphilic phospholipid shell allows the loading of amphiphilic compounds. The amino acid residues exposed by ApoB-100 can covalently bind to the therapeutic or diagnostic cargo. In brief, there are three methods for cargo carrying in LDL:^16^ loading in the hydrophobic core (Fig. 3A), inserting into the phospholipid monolayer (Fig. 3B), and binding to the apolipoprotein (Fig. 3C).

#### 3.1.1 Loading in the hydrophobic core

This method recombines exogenous lipophilic compounds into the LDL's non-polar cholesteryl ester core. This strategy was first proposed by Krieger *et al.* in the 1980s.^50^ The endogenous lipid core of LDL can be extracted and removed with non-polar organic solvents (such as heptane, benzene, or toluene) without damaging the integrity of the phospholipid and protein shell. During this process, this nanocomplex spontaneously recombines, and the recovery of LDLR binding activity exceeds 50%.^46^ We can also remove the endogenous lipid core through sonication or cholate dialysis.^51^ Each natural LDL particle can contain about 1200–1300 cholesterol esters and 250–300 triglycerides.^52^ Therefore, this method is suitable for transporting large numbers of hydrophobic molecules. These cholesterol-conjugated or -mixed compounds that mimic natural cholesterol esters divide into LDL in a physiological environment, enter tumor cells through receptor-mediated endocytosis, and are degraded, thereby releasing the coupling to play a diagnostic or therapeutic role.^53^ This core loading strategy can solve the problem of poor water solubility of diagnostic or therapeutic compounds and significantly increase their bioavailability.

#### 3.1.2 Inserting into the phospholipid monolayer

In this method, the payload is inserted into the phospholipid monolayer in a non-covalently bound manner. The payloads usually have a certain degree of amphiphilicity that allows their hydrophobic tails to be embedded into the phospholipid shell and the hydrophilic heads to be exposed to the surrounding water environment. The hydrophobic tail's embeddedness in the surface of LDL depends on the interaction force with the phospholipid layer (such as the van der Waals force). The portion that extends into the watery environment produces hydrogen bonds or ion interactions.^11,54^ The balance and strength of these forces often determine the stability and efficiency of loading. Surface loading has enabled the preparation of various LDL-based nanoformulations for diagnosis or therapy. Although this method is easy to implement, the leakage rate is relatively high. From a thermodynamic point of view, the surface loading is easy to dissociate from the LDL and translocate to the cell membrane surface.^46^

#### 3.1.3 Binding to the apolipoprotein

Protein loading is achieved by covalently binding the probe to the side chain of the apolipoprotein. Typical protein-binding points include lysine, arginine, tyrosine, and cysteine.^55^ ApoB-100 contains 357 lysine amino groups in the side chain, of which 225 are exposed on the surface. Among the exposed lysine residues, 53 were active lysine amino groups (p*K*_a_ 8.9), and the rest showed normal p*K*_a_ (10.5) and normal reactivity.^56^ Since less than 20% of lysine has binding activity, the small loading capacity of this type of loading is understandable. In addition, although covalent bond formation can stabilize the product, it also leads to the irreversible inactivation of ApoB-100.^46^ In other words, this covalent modification strategy may alter LDL's delivery properties. After all, the active binding site of LDLR is also located in ApoB-100. The ideal lysine residue modification should retain the binding ability to LDLR. In this regard, some scholars have explored ways to change LDL targeting by introducing new targeting ligands.^57^

### 3.2 Advantages of LDL-mediated delivery

As a versatile nanoscale building block, LDL has many unique advantages over other nanocarriers.^31,58–60^ First, LDL is a natural biological component with inherent biocompatibility, biodegradability, and non-immunogenicity. It is synthesized and metabolized in organisms and finally degraded into recyclable units, including cholesterol, fatty acids, and amino acids. In addition, LDL was able to evade the recognition of *in vivo* mononuclear phagocytes and reticuloendothelial systems, and the rapid clearance by the kidney due to the endogenous characteristics. Second, LDL has a long half-life (lasting 2–4 days). The long circulation characteristic of blood is conducive to the systemic delivery of drugs or probes. Third, the diameter of LDL is less than 30 nm in the nanometer range, making it diffuse from the blood vessel to the outside. The suitable size leads to good penetration of LDL through the interfibrillar opening (<40 nm) in solid tumors. Fourth, the primary defect of the widely used humanized targeting vectors, including monoclonal antibodies, growth factors, and hormones, is the low efficiency of cell internalization, which the LDL-mediated targeted delivery system can avoid. Furthermore, because of the LDLR over-expression in the tumor microenvironment, the selective affinity of LDL enhances the cellular uptake. Finally, LDL has a high loading capacity and can be processed by various loading strategies (specific methods will be described in the next section). The sizeable hydrophobic core allows LDL to functionally deliver hydrophobic bioactive compounds without changing the protein integrity and can effectively avoid the degradation or destruction of the load in the blood circulation. Amphiphilic shells are also well suited for loading amphiphilic compounds. In conclusion, LDL's size, structure, and various characteristics make it the preferred target vector.

### 3.3 Defects of LDL-mediated delivery

Although LDL-based nanocarriers show a series of advantages, some defects still limit the delivery process:^61^ (1) LDL is challenging to obtain and extract in large quantities, and the composition and size of different batches are variable. (2) LDL particles are isolated from human blood, so there is a pathogen risk of causing infectious diseases. (3) As a lipoprotein, LDL has poor storage stability and is easily degraded by *in vitro* physical or chemical factors. (4) Although LDL is an effective carrier for the targeted delivery of drugs and diagnostic agents to tumor sites, its application in cancer treatment and diagnosis is limited by the expression level of LDLR. (5) Like other nanocarrier systems, LDL also has the defects of limited drug encapsulation efficiency and surface loading capacity. (6) LDL cholesterol is considered to be associated with myocardial infarction and atherosclerotic cardiovascular disease.^62^ Therefore, choosing LDL as a targeted delivery carrier to deliver into the body is controversial.

### 3.4 Strategies on recombinant LDL

Because the defects of natural LDL limited the delivery applications, researchers tried to develop improved recombinant LDLs (rLDL). Recombinant lipoproteins are one of the most widely studied synthetic lipoproteins. Unlike natural lipoproteins extracted directly from human blood, recombinant lipoproteins are formed by combining isolated apolipoproteins with lipids (natural or synthetic analogs). Typically, each lipoprotein contains a single apolipoprotein type, while the lipid component type can be one or more.

Based on the known functions of natural LDL, rLDL has unique advantages and performances. First, since all compounds constituting rLDL are clear, it is easier to characterize and repeat according to their respective structural components. Secondly, the synthesis of rLDL is achieved by covalent derivatization or other chemical modification of the lipid and protein components of nanoparticles.^63^ It means that the physicochemical properties of LDL (size, zeta potential, core, and surface loading) can be flexibly controlled by separately adjusting the stoichiometric ratio of lipid/protein, the types of lipid, apolipoprotein, or other components. Studies have shown that increasing the proportion of protein to lipids tends to reduce the diameter of lipoprotein complexes.^64,65^ Other factors, such as the type of apolipoprotein, also influence the final size of complexes.^66^

As early as the 1860 s, Scanu *et al.*^67^ separated human apolipoprotein and phospholipid from donors by a two-step method (ultra-centrifugation density centrifugation and organic solvent extraction). Then they induced apolipoprotein and phospholipid to self-assemble to recombinant lipoproteins through lyophilization, solubilization, co-incubation, and other operations. Walsh *et al.*^68^ proposed using ApoB isolated from human plasma and combining it with phospholipids to form rLDL by using sodium deoxycholate. Subsequently, Ginsburg *et al.*^69^ and Lundberg *et al.*^70^ modified the method and successfully synthesized rLDL nanoparticles.

**Fig. 4:**
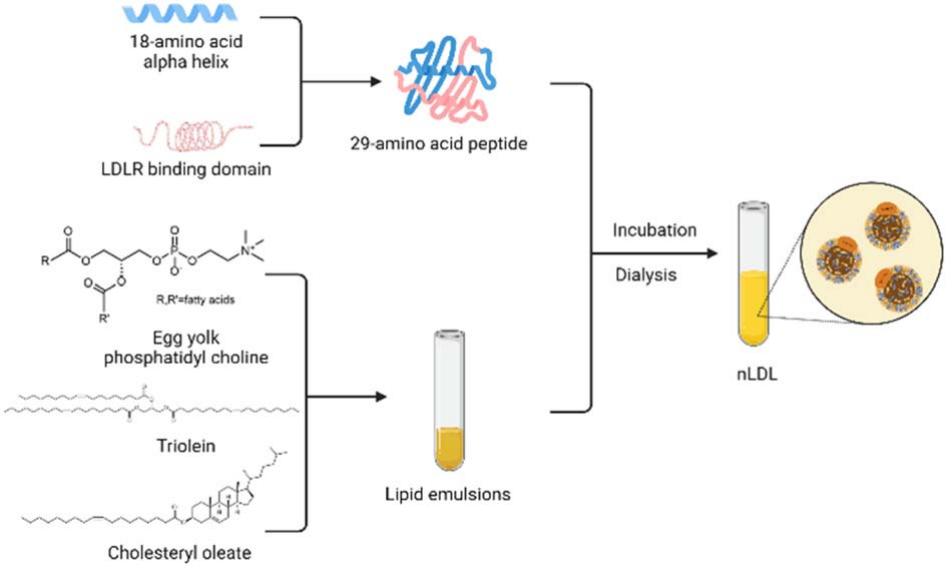
Strategy for synthesizing nLDL.

However, there is no denying that the development of rLDL preparations is plagued by the limited availability of ApoB-100.^51^ Over the years, researchers have made various attempts to overcome this obstacle. Law *et al.*^71^ successfully cloned human ApoB-100 into an expression vector and obtained 560 amino acid sequences of the protein after specific screening and identification. Moreover, methods to construct lipoprotein-like nanoparticles using peptides that mimic the functional properties of apolipoproteins have been reported many times. This technology enables the mass production of lipoproteins and accelerates clinical translation for the targeted delivery of recombinant lipoproteins. In 2002, Baillie *et al.*^72^ developed a synthetic LDL (sLDL) formulation using a combination of a lipid emulsion and an amphipathic peptide containing the ApoB receptor domain. sLDLs mimic natural LDL and can specifically bind to U937 tumor cells through LDLR. In 2007, Nikanjam and colleagues^27,73^ synthesized an amphiphilicity α Helical peptide, composed of 29 amino acids and containing a particular sequence with nine amino acids that LDLR can recognize. This functional peptide was used to prepare a nano-LDL (nLDL) through a microemulsion with phosphatidylcholine, triolein, and cholesteryl oleate (Fig. 4). The study also demonstrated that the nLDL vector could target GBM cells with high LDLR expression. In the subsequent research, they loaded Paclitaxel Oleate (PO) into the core of nLDL to prepare drug-loaded nanocapsular-PO. The LDLR selectivity of nLDL shows the potential for targeted drug delivery as a vehicle. The advantages of peptide segments to simulate apolipoproteins are in many aspects, such as purity, quantity, processing time, and safety.^74^ Therefore, this method of synthesizing lipoproteins has received extensive attention from researchers.

## 4. LDL-mediated targeted delivery

### 4.1 Delivery for diagnosis

**Fig. 5:**
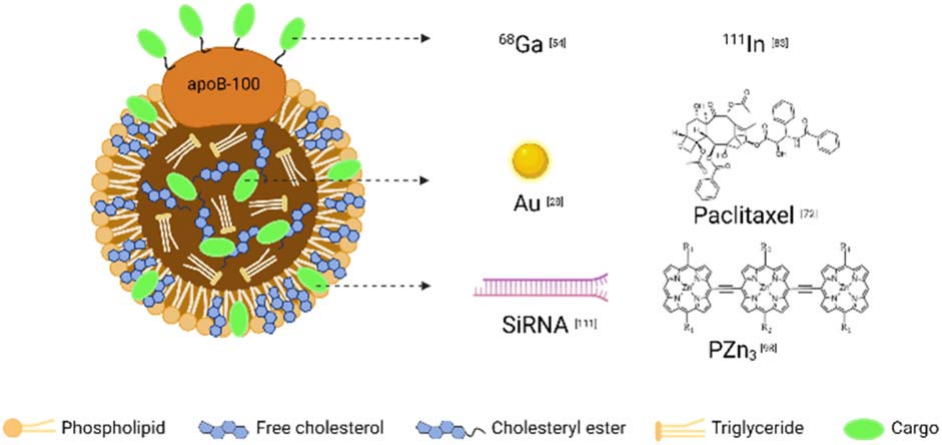
Representative cargoes in the LDL-mediated targeted delivery system for diagnosis and therapy.

Combining different contrast agents allows LDL to have additional tumor imaging capabilities. We summarized the examples in Table 2 and showed the representative diagnostic cargoes in Fig. 5.

**Table 2:** Summary of examples of low-density lipoproteins for imaging

Contrast agent type	Examples	Ref.
Radioactive tracers	^99m^Tc	74–76
	^125^I, ^131^I	77–81 and 87
	^68^Ga	54
	^111^In	83
CT	ITG	88
	Au	28
MRI	Gd^3+^	90
	Gd-AAZTAC17	37
Fluorescent dyes	TCL	97
	PZn_3_	98
	Pz 247	99
	Dil	100
	DiR	45
	SWIR-WAZABY-01	101

#### 4.1.1 Loading radioactive tracers

*In vivo* imaging and characterization of LDL labeled with a radioactive tracer can be traced back to more than 30 years. Among them, technetium-99m (^99m^Tc), iodine-125 (^125^I), and iodine-131 (^131^I) are the most commonly studied and used radiolabels *in vivo* imaging. Lees *et al.*^75^ first used ^99m^Tc as a labeling agent for LDL and covalently linked ^99m^Tc with LDL in the presence of a reducing agent. The results showed that ^99m^Tc LDL could be used for *in vitro* imaging to evaluate the organ distribution of LDL in rabbits without significantly changing LDL's *in vivo* metabolic mode. They successfully detected the accumulation of ^99m^Tc LDL in human atherosclerotic plaque by using a gamma scintillation camera.^76^ Isaacsohn *et al.*^77^ demonstrated for the first time that ^99m^Tc labeled LDL can be used to assess adrenal cortical function through external imaging.

The experimental results of labeling LDL with iodine monochloride technology showed that the combination of ^l25^I and LDL was stable.^78^ Ultrastructural autoradiography can be used to examine the effect of atherosclerotic regions of blood vessels on lipoprotein uptake and localization. Lewis and colleagues^79^ preferred white Carneau pigeons 0.25% cholesterol-supplemented diet to accelerate atherosclerosis in an experiment. After successful modeling, homologous ^125^I-LDL was injected intravenously, and the pigeons were killed one hour later. Autoradiography showed rapid absorption of ^125^I-LDL, which accumulated mainly in the liver, followed by the lung, kidney, spleen, and aorta. Compared with ^125^I, ^131^I has a shorter half-life but higher radiation energy. Therefore, ^125^I and ^131^I are widely used as radiolabels for various lipoproteins *in vitro* and *in vivo*.^80–82^ However, iodized LDL samples can cause lipid peroxidation and change the biological characteristics of LDL, and the most obvious consequence is to reduce its specific binding ability to LDLR. The immediate introduction of butylated hydroxytoluene and ascorbic acid can solve this problem. Studies have shown that these two antioxidants significantly inhibit radioiodine-induced lipid peroxidation and LDL modification.^83^ In addition, LDL labeled with gallium-68 (^68^Ga) or indium-111 (^111^In) has been considered as potential radioactive markers for positron emission tomography (PET) or single-photon emission computed tomography (SPECT) imaging.^54,84^

The method of immobilizing radionuclides directly on the surface of LDL has been reported in several studies. It has also been proposed to connect the radionuclide to apolipoprotein through the cyclic anhydride of diethylenetriaminepentaacetic acid (DTPA) to achieve chelation. However, this bifunctional agent will cause intramolecular or intermolecular crosslinking, leading to ApoB-100 deactivating.^85,86^ Therefore, some scholars chose to incorporate the lipophilic derivative of DTPA into the phospholipid monolayer of LDL to reduce the structural modification of ApoB-100.^54^ Nevertheless, these radiolabeling methods are inevitably limited by the degradable nature of lipoproteins. Once LDL is degraded in the body, radioactive markers will quickly leak and redistribute.^87^ So this process cannot quantitatively reflect the catabolism in tissues. The LDL-labeled residualized moiety tyramine-cellobiose can solve this difficulty. Because even when lipoproteins are metabolically degraded, tracers remain in tissue cells.^84^ In addition, Xiao *et al.*^88^ synthesized an (125)I-labeled hexa-iodinated diglyceride analog, named 1,3-dihydroxypropan-2-one 1,3-diiopanoate (DPIP). LDL is labeled with core reconstitution and is resistant to lysosomal degradation after cellular uptake.

#### 4.1.2 Loading CT and MRI contrast agents

Although PET and SPECT have excellent sensitivity in the picomolar range, the resolution of nuclide imaging is limited. In contrast, the spatial resolution of computed tomography (CT) is much higher. Therefore, several studies have explored the targeting mechanism of LDL using CT imaging.

Because of the polarity of conventional X-ray agents, linking these molecules to LDL is more complicated than radionuclides. Hill *et al.*^89^ explored the incorporation of poly-iodinated triglyceride (ITG) into LDL for targeted delivery of CT contrast agents. They first developed an ITG-loaded LDL ((rITG)LDL). In the cell particle CT imaging, the attenuation was weak with excessive natural LDL, which indicated that excessive natural LDL effectively inhibited (rITG)LDL from entering human hepatoblastoma G2 (HepG2) cells, further confirming that LDL modified with ITG did not damage its ability to recognize LDLR and interact with it. Allijn *et al.*^28^ established a new LDL labeling method based on sonication and ultracentrifugation purification. Specifically, the method first encapsulated various payloads (such as gold nanoparticles and lipophilic or amphiphilic fluorophores) in micelles, and then transferred them to the LDL core by sonication and ultracentrifugation. The experimental results *in vitro* and *in vivo* showed that LDL loaded with gold nanocrystals had similar characteristics in size, morphology, composition, oxidation state, ApoB-100 function, and molecular weight compared with untreated natural LDL. In conclusion, as one of the few reports on targeted CT contrast agents, Au-LDL can be used as a marker to study LDL interaction, cholesterol metabolism, atherosclerosis, tumor growth, and other fields.

Since magnetic resonance imaging (MRI) has the advantages of high spatial resolution, no radiation,^90^ and no depth limitation, LDL has also been explored for the delivery of MRI contrast agents. The most commonly used MRI contrast agents in clinical practice are the paramagnetic chelate of Gd^3+^ ions to increase the longitudinal relaxation rate of water protons distributed in tissues. Corbin *et al.*^91^ inserted amphiphilic Gd-DTPA chelate into a LDL phospholipid monolayer to achieve surface modification of LDL. The results of the structural and functional properties of Gd-labeled LDL nanoparticles showed a similar diameter and surface charge, and the ability to bind to LDLR as native LDL. In addition, LDL-containing tissues (liver and HepG2 tumors) showed significant MRI contrast enhancement after intravenous administration of Gd-labeled LDL. Thus, Gd-labeled LDL has the potential to be an MRI contrast agent for *in vivo* tumor detection. However, the disadvantage of this targeting system is its relatively low relaxation. On this basis, to enhance the relaxation of the complex system and improve the thermodynamic stability, Crich and colleagues^37^ developed a new Gd complex, namely Gd-AAZTAC17/LDL. When added to the incubation medium, the Gd-AAZTAC17/LDL adduct was absorbed by HepG2 and melanoma B16 tumor cells. *In vivo* MRI analysis of C57BL/6 mice transplanted with melanoma B16 cells showed that the enhancement of tumor signal intensity 8 hours after the injection of the Gd-AAZTAC17/LDL adduct was significantly higher than that of Gd-AAZTAC17 alone. Therefore, this Gd complex can be an effective and sensitive MRI probe. Several LDL-based MRI probes have been reported in recent years after the optimization of the targeting system.^92–96^

#### 4.1.3 Loading fluorescent dyes

Near-infrared fluorescence (NIRF) imaging has been proven to be a non-invasive and effective real-time imaging method.^97^ Therefore, applying LDL probes based on NIRF imaging in medical diagnosis has been developing for a long time.

As early as the early 2000s, Zheng *et al.*^98^ synthesized a tricarbocyanine cholesteryl laurate (TCL) with primary amine functional groups. A stable conjugate probe TCL17-LDL was prepared by chelating TCL to the lipid region of LDL. TCL17-LDL was i.v. injected to detect HepG2 tumor tissues by using a low-temperature 3-D redox scanner. Images showed an enhanced fluorescent signal only for the tumor tissue, confirming the tumor-targeted delivery of TCL17-LDL. Wu *et al.*^99^ incorporated meso-to-meso ethyne-bridged tris [(porphinato) zinc(ii)] (PZn_3_) into the hydrophobic core of LDL. *In vitro* experiments demonstrated that PZn_3_-containing LDL transferred core NIRFs to murine B16 melanoma cells through the LDLR pathway and achieved the imaging of B16 cells by confocal near-infrared fluorescence microscopy in a shallow dose (∼nM). Trivedi *et al.*^100^ reported the synthesis of chiral porphyrazine (pz), H2 [pz (*trans*-A2B2)]-247 (Pz 247). Pz 247 bound to the lipophilic core of LDL and entered the cell primarily through receptor-mediated endocytosis. *In vivo* studies have shown that Pz 247 exhibited preferential accumulation and retention in murine subcutaneously implanted MDA-MB-231 tumor cells, thus enabling NIRF imaging to distinguish tumors from surrounding normal tissue.

As a classic carbocyanine dye, 1,1′-dioctadecyl-3,3,3′,3′-tetramethylindocarbocyanine perchlorate (Dil) was used to label native or modified LDL. Li and colleagues^101^ synthesized and validated the possibility of noninvasive NIRF imaging of LDLR-overexpressing tumors using Dil-labeled LDL. In addition, they confirmed the selective accumulation of DiI-LDL in the tumor area using confocal microscopy and three-dimensional cryo-imaging.

Unlike the above targeting methods, Chen *et al.*^45^ introduced an LDL redirection strategy. The strategy was to transfer LDL nanoparticles from their native receptor to alternative surface receptors or epitopes by coupling tumor-directed ligands to active lysine residues exposed in ApoB-100. Following this idea, they prepared LDL nanoparticles labeled with the fluorescent dye 1,1′-dioctadecyl-3,3,3′,3′-tetramethylindotricarbocyanine iodide (DiR) and modified with folic acid (FA), DIR-LDL-FA. The probe was accumulated in KB cells for the folate receptor (FR) or LDLR overexpression but only slightly absorbed in cells without FR (CHO and HT1080).

To solve the problems of long-term toxicities, low quantum yields, and poor water solubilities of some NIR-II fluorophores, Kalot *et al.*^102^ developed a water-soluble NIR-II emitting nitrogen BODIPY derivative SWIR-W AZABY-01. They used lipoprotein gel electrophoresis and ultracentrifugation to prove that the SWIR-WAZABy-01 fluorophore mainly interacted with LDL in human plasma. LDL drove the circulation of SWIR-WAZABy-01 in the blood and strongly increased its fluorescence emission. Therefore, SWIR-W AZABY-01 could be used as a new and relevant tool to label LDL for pathology effectively.

### 4.2 Delivery for therapy

#### 4.2.1 Loading therapeutic agents

As mentioned before, because large amounts of lipoproteins are required for rapid tumor proliferation, many malignancies increase LDL acquisition by upregulating LDLR.^31^ Therefore, the use of compounds that combine various drugs with LDL can induce tumor cell death at target sites.^103^ Compared with free drugs, drug-loaded LDL can target tumor cells more precisely and efficiently, significantly reducing the side effects caused by drugs in non-tumor sites. Therefore, many reports have been on using LDL as an anticancer drug carrier for cancer treatment (Table 3). We showed the representative therapeutic cargoes in Fig. 5.

**Table 3:** Summary of examples of low-density lipoproteins for therapy

Load type	Examples	Ref.
Therapeutic agents	DOX	103–106
	Paclitaxel	72 and 107–109
	siRNA	110–114
	DHA	117–121
	5-Fluorouracil	122
	Imatinib	123
	Donepezil	25
	Oxaliplatin	124
	Thiosemicarbazone metal–ligand complexes	125
	Polysaccharide	126
	Dexamethasone palmitate	130–132
Photosensitizers	Porphyrin compounds and derivatives	135, 137 and 138
	Pyropheophorbide cholesterol oleate conjugate	139
	SiPcBOA	140
	SiNcBOA	141
	Bchl-BOA	142
	TPA DPPy	143

Chu *et al.*^104^ conjugated the cytotoxic drug doxorubicin (DOX) to human LDL to form a complex (LDL-DOX). When injected into mice, LDL-DOX accumulated more in the liver than free DOX and less in the heart. Both histological and enzymatic analyses showed that LDL-DOX could reduce DOX-induced cardiotoxicity. Over the next decade, various DOX-loaded LDLs were designed and used to explore the treatment of different tumor models. For example, Pinzón-Daza and colleagues^105^ combined statins with DOX-loaded LDL to convert a less permeable DOX into a drug capable of crossing the blood–brain barrier (BBB), thereby achieving significant anti-brain tumor cytotoxicity. Tian and colleagues^106^ encapsulated DOX with self-assembled nanoparticles composed of chitosan and LDL. After the incubation of this pellet with gastric cancer SGC7901 cells for 24 h, encapsulated DOX showed higher cellular uptake than free DOX. A recent study reported a PH-sensitive ApoB-100/Oleic-DOX/NLC (AODN) nanoparticle based on a nanostructured lipid carrier. The experimental results show that AODN nanoparticles can accumulate more drugs in the tumor site, reduce systemic toxicity, and effectively inhibit *in situ* breast cancer.^107^

LDL is also reported to be used by researchers to load another common cytotoxic drug, paclitaxel. Nikanjam and coworkers^73^ incorporated the lipophilic prodrug paclitaxel oleate into the lipid core of LDL particles to prepare drug-loaded particles that could target and kill glioblastoma multiforme cells. Unlike this, another glioma treatment study^108^ developed nanoparticles loaded with dual-targeting paclitaxel by modification with peptide-22 (PNP-PTX). Su *et al.* designed a sLDL to encapsulate paclitaxel-alpha linolenic acid (PALA). PALA-loaded sLDL (PALA-sLDL) had higher tumor accumulation and tumor suppression efficiency than a PALA-loaded microemulsion (PALA-ME, without the LDLR binding domain). In addition, the use of paclitaxel-loaded LDL in lung cancer models has been reported. Kim *et al.*^109^ developed a solid lipid nanoparticle (SLN) with paclitaxel at its core. Qian *et al.*^110^ used lipoprotein-mimic nanoparticles modified with an amphiphilic hybrid peptide to deliver paclitaxel.

Interestingly, neither of these studies directly utilized LDL but developed biomimetic LDL nanocarriers instead. Nevertheless, both LDL-like agents showed significant antitumor activity in lung cancer models, demonstrating the potential use of these nanocarriers to improve therapeutic efficacy and reduce the side effects of antitumor drugs.

Small interfering RNA (siRNA) plays a vital role in silencing multidrug resistance genes in tumors.^111^ Therefore, developing effective and stable siRNA vectors is crucial for siRNA system delivery. Zhu and coworkers^112^ loaded cholesterol-binding siRNA onto LDL, and DOX-loaded chol-siRNA/LDL-coupled *N*-succinyl chitosan nanoparticles (Dox-siRNA/LDL-SCS-NPs) were prepared and characterized. *In vivo* tumor targeting demonstrated significant accumulation of this agent in an orthotopic liver tumor model. Jin and colleagues^113^ combined SLN with pegylated c-Met siRNA. This complex not only effectively down-regulated the expression level of c-Met *in vitro* but also reduced cell proliferation in U-87 MG. Yang *et al.*^114^ developed a new micelle loaded with paclitaxel and an LDL nanoparticle loaded with siRNA, which were coupled to form a “binary polymer”. For cancer cell targeting, this system has the potential to be used to co-deliver siRNA and antitumor drugs to address multidrug resistance in cancer. Notably, in one study,^115^ siRNA was also used to target connective tissue growth factors to treat liver fibrosis. This study showed that the stable nanocomplex could be precisely delivered to the liver and silence the targeted gene by cell internalization. It reduced the collagen content and pro-fibrogenic factors and significantly improved the pathophysiological symptoms of liver fibrosis model rats.

As a natural omega-3 polyunsaturated fatty acid, docosahexaenoic acid (DHA) has antitumor properties.^116,117^ Reynolds and colleagues^118^ investigated the cytotoxic effects of DHA-loaded LDL nanoparticles (LDL-DHA) on the liver. The therapeutic utility of LDL-DHA nanoparticles was evaluated in normal and malignant murine liver cell lines TIB-73 and TIB-75, respectively. After administration, therapeutic doses of LDL-DHA ultimately killed TIB-75 but did not harm TIB-73. This study demonstrated the potential of LDL-DHA nanoparticles as an anti-hepatocellular carcinoma agent. On this basis, scholars have continuously verified the effect of LDL nanoparticles loaded with DHA on liver tumors.^119,120^ A study also explored the molecular mechanism underlying the anticancer activity of LDL-DHA nanoparticles and indicated that LDL-DHA could induce HCC cell death through the iron-death pathway.^121^ Additionally, DHA also plays a role in regulating the neural function of the brain. Mulik and colleagues^122^ then used pulsed focused ultrasound (FUS) exposures to open up the BBB to deliver LDL-DHA locally to the brain. FUS exposures resulted in a 2-fold increase in DHA levels in target brain regions compared with nontarget regions or control treatment groups. This technique provides an alternative idea for targeting acute brain injury areas or invasive tumor cells in the brain.

In addition to these well-studied anticancer agents, other anticancer drugs such as 5-fluorouracil,^123^ imatinib^124^ donepezil,^25^ and oxaliplatin^125^ have also been explored to be loaded onto LDL for targeted delivery. In a recent study,^126^ the anticancer agent thiosemicarbazone metal–ligand complexes were encapsulated in LDL, targeting breast (MCF7), lung (A549), and prostate (C42) cancer cells, respectively. Cytotoxicity assessment reported effective cancer cell growth inhibition in all studied cell lines. In addition, western blot analysis showed that tubulin expression was significantly reduced when the cell line was treated with LDL encapsulated with thiosemicarbazone metal–ligand complexes. In conclusion, all the experimental results indicated the potential feasibility of LDL as an active drug delivery strategy for cancer treatment.

Interestingly, all the above studies used the targeted delivery method of intravenous administration, while Zhou *et al.*^127^ took a different approach and explored a novel oral delivery modality. They combined a polysaccharide with LDL (extracted from fresh egg yolk) to form a composite nanogel with a diameter of less than 85 nm, which was able to encapsulate curcumin. Under simulated gastrointestinal conditions, polysaccharides can achieve stable and sustained release of curcumin while protecting LDL from enzymatic degradation in the stomach. This study suggests that LDL/polysaccharides could be a potential oral delivery system for drugs or nutrients.

It is important to note that LDL can also be used as an anti-atherogenic drug carrier. When LDL in the aorta is continuously deposited and oxidized, it will be converted into foam cells after engulfing by the recruited macrophages, thus intensifying the formation of atherosclerotic plaque. Compared with natural LDL, those oxidized particles have a higher affinity for atherosclerotic plaque. Therefore, LDL can be used as an ideal carrier for the specific delivery of drugs to atherosclerotic lesions.^128–130^ Tauchi and colleagues^131–133^ focused on this exploration and reported several studies. By incorporating dexamethasone palmitate (DP) into LDL, they demonstrated the inhibitory effect of the DP-LDL complex on foam cell formation through serial *in vivo* and *in vitro* experiments. They also examined the effect of DP-LDL on cholesteryl ester accumulation in the aorta of atherosclerotic mice. The DP-LDL complex showed a 100-fold decreased cholesteryl ester accumulation in the aorta than the free dexamethasone. Although more long-term studies are needed to establish validation, the DP-LDL complex is expected to guide the treatment of atherosclerosis in the clinic.

#### 4.2.2 Loading photosensitizers

Photodynamic therapy (PDT) is a promising way to treat cancer. This therapy requires the systemic application of a photosensitizer (PS) followed by local irradiation at a specific wavelength corresponding to the absorption band of PS. In this process, the increased production of reactive oxygen species leads to oxidative stress, which induces tumor cell death for therapeutic effects.^134^ But most PSs are hydrophobic or amphipathic and cannot be administered intravenously. Endogenous LDL is an attractive carrier option to improve the water solubility and tumor targeting of these PSs. LDL plays a vital role in the blood transport of highly hydrophobic PS.^135^ It has been shown that the binding of LDL to PS may promote their specific delivery to tumor cells, thereby increasing the efficacy of PDT.^136^

Moreover, another advantage of using LDL during PDT is that LDL is highly oxidized after irradiation. The resulting oxide is cytotoxic to endothelial cells and can further prolong the photodynamic effect.^137^ The examples mentioned below can be found in Table 3.

Among all photosensitizers, porphyrin compounds and their derivatives have been widely used in cancer PDT due to their unique advantages, such as high singlet oxygen quantum yields, apparent lack of dark toxicity, and strong adsorption capacity in the near-infrared region.^39^ For example, haematoporphyrin,^138^ benzoporphyrin derivative,^136^ tetraphenylporphyrin,^139^ and other porphyrin-related PSs incorporated into LDL for PDT have been reported earlier.

Earlier studies focused on the non-covalent binding of PS to LDL or the covalent ligation of PS to ApoB-100. However, Zheng and colleagues concentrated on core-load reconstruction strategies to improve the labeling rate of PS and serum stability. In one study, they synthesized a pyropheophorbide cholesterol oleate conjugate and successfully reconstituted it into the lipid core of LDL. Laser scanning confocal microscopy showed that this rLDL could be internalized into HepG2 tumor cells *via* the LDLR pathway.^140^ Later, they^141^ designed SiPcBOA, a tetra-*t*-butyl silicon phthalocyanine compound with two oleate moieties in the axial position. The final synthesized rLDL(r-SiPcBOA-LDL) had a very high payload (SiPcBOA to LDL molar ratio > 3000 to 35 001 : 1). Similarly, they^142^ synthesized a novel NIRF imaging agent tetra-*t*-butyl silicon naphthalocyanine bisoleate (SiNcBOA). Again, high loading (100 : 1 payload) was achieved while successfully reassembling it into the LDL lipid core. Besides, they^143^ engineered reconstituted bacteriochlorin e6 bisoleate LDL (r-Bchl-BOA-LDL) and evaluated the efficacy of PDT using a mice tumor model. Compared with the control group, the group injected with 2 μm per kg r-Bchl-BOA-LDL significantly delayed tumor regeneration.

Recently, Wang and coworkers^144^ reported for the first time that natural LDL particles were recombined with saturated fatty acids and mitochondria-targeting aggregation-induced emission (AIE) PSs. The new AIE photosensitizer (TPA DPPy) encapsulated in LDL granules was effectively absorbed by cancer cells and released to the mitochondria. Under light irradiation, reactive oxygen species produced around mitochondria lead to irreversible apoptosis of cancer cells. Unlike previous studies, this process can be monitored in real time by fluorescence, such as significantly enhanced luminescence and blue-shifted emission. This innovative method of providing real-time fluorescence feedback on treatment results has dramatically improved the quality of PDT.

## 5. Conclusions and prospects

Since lipoproteins are genius drug carriers and diagnostic tools, the research of lipoprotein-based nano-delivery systems has been carried out and developed continuously. Among them, LDL, as a prominent representative, has increasingly diversified applications in the field of targeted delivery. LDL-based carriers can transport various substances and reagents, including imaging agents, chemotherapeutic drugs, antiviral drugs, antibacterial agents, and siRNA. These applications provide great potential for clinical transformation. LDL-based nanoparticles show a series of advantages, such as superior payload capacity, long residence time in circulation, excellent biocompatibility, smaller size, and natural targeting. However, there are still many problems to be solved. The difficulty in obtaining and purifying natural LDL is one of the main reasons that limit its research and application. The functionalized rLDL provides a new direction for applying LDL as a carrier. Exploring the construction of novel lipoproteins or apolipoproteins can offer novel ideas and methods for designing drug delivery systems. It is affirmative that novel LDL-based nanoformulations will become a promising diagnostic and therapeutic drug delivery nano-platform. However, more systematic and comprehensive *in vivo* evaluation is necessary to safely use LDL preparations in humans. Admittedly, LDL and its derivatives, driven by advances in nanotechnology and bionics, will play a key role in solving more human diseases.

## Conflicts of interest

There are no conflicts to declare.

## Supplementary Material
